# Improving the staging of neck injuries using a new index, the Neck Functional Holistic Analysis Score: Clustering approach to determine degrees of impairment

**DOI:** 10.1371/journal.pone.0238424

**Published:** 2020-09-09

**Authors:** Alberto J. Fidalgo-Herrera, Carlos Jové-Blanco, Mª Jesús Martínez-Beltrán, José A. Moreno-Ruiz, Julio C. de la Torre-Montero

**Affiliations:** 1 Biomechanics Department, Fisi(ON) Health Group, RetiaTech, Las Rozas, Spain; 2 San Juan de Dios School of Nursing and Physical Therapy, Comillas Pontifical University, Madrid, Spain; 3 Department of Informatics and Computation, University of Almería, Almería, Spain; University of Manitoba Faculty of Health Sciences, CANADA

## Abstract

**Background:**

Traumatic cervical spine injuries are amongst the traffic injuries that can cause most harm to a person. Classifying subtypes of clinical presentations has been a method used in other pathologies to diagnose more efficiently and to address the appropriate treatment and the prognosis. The management of patients suffering from cervical injuries could be improved by classifying the severity of the impairment. This will allow clinicians to propose better treatment modalities according to the severity of the injury.

**Materials and methods:**

The present study is a retrospective cohort study performed with the clinical data from 772 patients stored at Fisi-(ON) Health Group. All the patients treated for cervical spine injuries are evaluated using the EBI-5^®^ system, which is based on inertial measurement unit (IMU) technology. The normalized range of motion of each patient was incorporated into a single index, the Neck Functional Holistic Analysis Score (NFHAS).

**Results:**

Clustering analysis of the patients according to their NFHAS resulted in five groups. The Kruskal-Wallis H test showed that there were statistically relevant differences in the ROM values and NFHAS of the patients depending on the cluster they were assigned to: FE X^2^(4) = 551.59, p = 0.0005; LB ROM X^2^(4) = 484.58, p = 0.0005; RT ROM X^2^(4) = 557.14, p = 0.0005; NFHAS X^2^(4) = 737.41, p = 0.0005. Effect size with ηp^2^ for the comparison of groups were: FE = 0.76, LB = 0.68, RT = 0.76 and NFHAS = 0.96.

**Conclusion:**

The NFHAS is directly correlated to the available ROM of the patient. The NFHAS serves as a good tool for the classification of cervical injury patients. The degree of impairment shown by the cervical injury can now be staged correctly using this new classification.

## Introduction

Traumatic cervical spine injuries are amongst the traffic injuries that can cause most harm to a person. Traumatisms to the cervical spine can potentially injury the spinal cord, cause respiratory disfunction or produce internal bleeding, all of which could be fatal to the patient. Most of neck injuries result from an acceleration / deceleration of the head because of the collision. The injury results from a whiplash mechanism that takes the following sequence: in the first 50 milliseconds (ms) the head moves directly back; between 50 and 75 ms the spine takes the shape of an "S", the upper part of the spine would be in flexion while the lower part would remain in extension; finally, from 75 to 100 ms the head goes to hyperextension in relation to the spine. The severity of the injury increases with the impact speed since the head experiences twice the deceleration of the car at the moment of impact [1]. Exhaustive attention is made to discard severe injuries, with different protocols such as the Canadian C-Spine, the Low risk criteria, and the flexion-extension radiography. All these protocols make relationships between the movement of the cervical spine and the integrity of the structure. However, discarding structural damage does not imply that no damage has been done to the cervical spine.

The ICD-11 includes many diagnostic terms to address cervical spine injuries [2]. The most identifiable pathology from traffic car accidents is Whiplash Associated Disorder (WAD). This injury exemplifies the presence of pathology in the absence of identifiable structural damage. A preliminary investigation in 2015 highlighted the possibility of having spinal cord injuries in subsamples of WAD patients [3], while others argue about the existence of morphologic and compositional changes in the neck muscles of WAD patients [4–6]. None of these findings are definitive and normally radiological signs are negative. The usual examination of this patients mainly involves visual examination and the evaluation of patient reported symptoms. Diagnosis, severity, and prognosis are established relying on subjective information gathered and reported by both the clinician and the patient, respectively.

Neck injuries pose a challenge for health professionals because of their high incidence (300 cases/100000 inhabitants per year), the absence of conclusive clinical tests, the controversial relationship with insurance compensations and the malingering [7, 8]. The given diagnosis is mainly based on the subjective complaints from the patient and the causal factor of being involved in a traffic collision [9]. Some studies indicate that almost 85% of injured people from car accidents report neck complaints after the accident [10, 11]. It has also been noted that the incidence of patients reporting neck injuries increases with decreasing severity of the collision [7].

Some attempts have been made to classify patients into degrees of injury such as the Quebec Task Force (QTF) classification for WAD. This grading is made relying on gross kinematic observations and reported symptomatology [12]. Grades 3 or 4 are easily identifiable because the first one implies the existence of neurological signs and the second one implies structural compromises. However, grades 1 and 2 are not easily distinguishable since it is difficult to draw a line that clearly separates stiffness from decreased range of motion (ROM) [13]. Visual examination demonstrated poor reliability for ROM assessment [14] and hand held goniometers do not provide good reliability either for the assessment of neck ROM [15]. Assigning a QTF grade 1 or 2 is quite an arbitrary decision based upon consensus rather than on the objective measurement of the ROM and the proper appraisal of what is a ROM limitation and what is not. Furthermore, decreased ROM in grade 2 is not further stratified. Mild reductions of ROM are being equated to severe reductions, while both situations can imply very different outcomes for the patient and handling difficulties for the health professional.

Other investigations have already used the kinematics for the subclassification of pain [16] or the identification of different types of abnormal movement patterns in a joint [17]. Classifying subtypes of clinical presentations can help to diagnose more efficiently, propose the best treatment and address prognosis. Subclassifying can highlight different disfunctions that used to be present under the same diagnostic label and allow for a full understanding of the problem [18]. This classification will give the best results if it is based on objective measures. With the increasing availability of cheap and reliable measuring devices based on inertial measurement units (IMU) and the advancement of health informatics, more professionals start to rely on objective evaluations [19]. Still, there is a lack of a clear consensus on which main variables from neck movement should be addressed on a basis [16, 20–25] and which is their association to the blunt trauma suffered [26]. Even the previous state of the cervical spine of the patient, difficult linking the trauma to the findings obtained [7]. Nevertheless, some investigators have been able to use kinematics to correctly differentiate patients suffering from WAD from others that do not [27]. As kinematics can differentiate healthy from injured patients, there could be also differences between the kinematics of the injured ones. Moreover, the progression of pain along treatment has shown to correlate with the progression of kinematics such as ROM [28]. A classification based on ROM impairment could help with the management of these patients since it could identify the ones with more severe symptoms.

According to the previous information, three main objectives will be sought in this research:

1. Evaluate if the different classical diagnoses show differences in the ROM of the included patients. Finding differences between the groups would show that different diagnoses are describing different degrees of injury.
2. Create an index that can describe the ROM of all the three planes of motion of the neck. This new index would help on the interpretation of the severity of ROM limitation and allow for a classification based on the degree of impairment.
3. Propose a classification based on the index created for the neck ROM if classical diagnoses do not cluster patients according to severity of ROM impairment.

## Methods

### Study design

The present research is a retrospective cohort study formulated with the clinical data from 772 patients. All the patients had suffered a traffic car accident and have been diagnosed with neck pathology. None of the included patients was still in treatment during the time this investigation was undertaken.

Personal data from the patients was anonymized from their medical records before the research team could work with the data. The principles of the Helsinki declaration were considered throughout the design and performance of the investigation. The project received ethics approval from *Hospital Clínico Universitario San Carlos*, *Madrid*, *España* (Spain), with identification code 18/405-E under the name “Estudio de datos cinemáticos de la columna cervical”.

This investigation has a single blind nature. The ROM measurements of the patients were performed by teams independent to the research team, no contact existed between them. All the teams were trained alike in the use of the EBI-5^®^, which is an IMU based system.

### Subjects

The inclusion criteria were: age over 18 years old, injuries due to a traffic car accident, diagnosis of a cervical spine pathology, evaluated between August and October 2018, measured with the EBI-5^®^ [29]. Only the initial ROM evaluation conducted before rehabilitation was selected. The exclusion criteria were: rehabilitation prior to ROM assessment, neurological signs or structural compromise of the neck.

Information from all patients in this investigation was comprised into the medical records of Fisi(ON) Health group, a provider of medical services. Convenience sampling was used. All the subjects had signed an informed consent form prior to evaluation and treatment. Patients in the database are from all over Spain, 90 possible measuring spots were available at the time.

**Fig 1:**
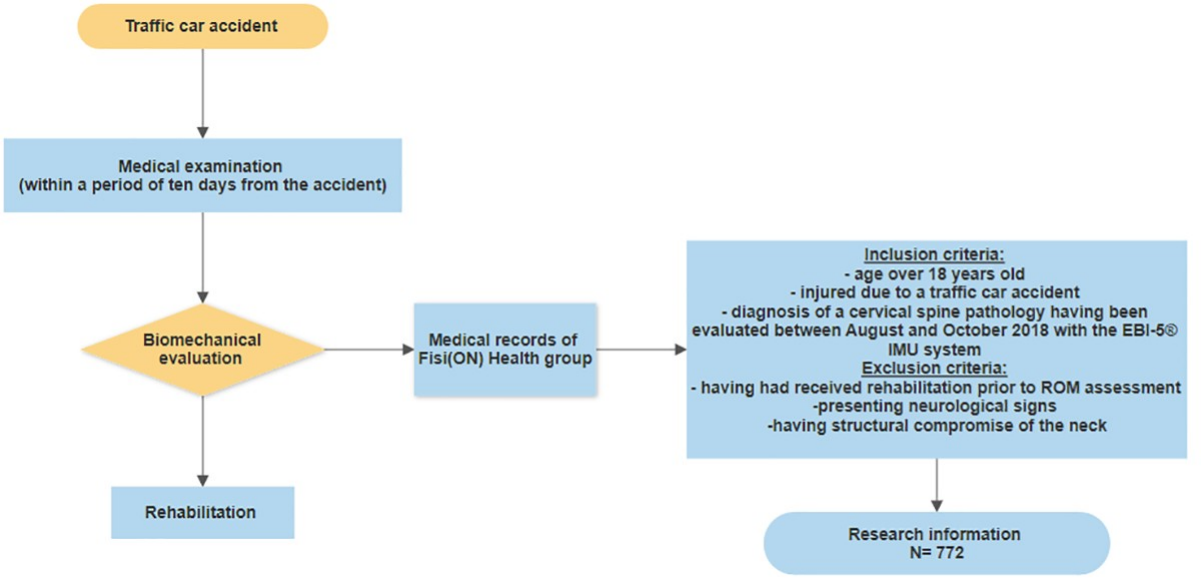
Flow of the subject through medical care and information recovery. The participants were examined by a medical doctor within 10 days of the accident. Biomechanical assessments were made and stored. The participant received normal rehabilitation. None of the data in this investigation came from active cases.

All the included patients followed the same steps through medical care provided by Fisi(ON) Health group. Within a period of ten days from the accident, the patients are appointed for medical examination. If the patients are diagnosed with a neck injury and rehabilitation is prescribed, they are appointed for biomechanical evaluation before starting the treatment. The information from that biomechanical assessment was gathered for this investigation. This flux is represented in Fig 1.

### Data acquisition and variables

The EBI-5^®^ is an accelerometry based system with a precision of ±0.1^o^ validated and independently tested by an audit from App^+^ metrological company. The device uses two IMU for the recordings. One is placed upon the occiput with an elastic headband, and the other one between the spinous processes of T2 and T3 with a double-sided hypoallergenic sticker. All participants perform the maximum number of repetitions in a period of 45 seconds for each plane of motion: flexion-extension (FE), lateral bending (LB) and rotations (RT). All motions are performed in an upright sitting position at a self-determined velocity. The selection of 45 seconds of measuring time allows for all patients to have enough time to perform enough number of repetitions of the movement to have a clear picture of their actual mobility. The patients can make the movements at their own pace depending on the degree of injury or age capability, without external interference to adapt to preset procedures.

Since the device uses two IMUs, all compensation movements are excluded from the recording. The measure given by the system is the difference between the measure of one IMU against the other.

To accomplish our objectives several variables were gathered. All variables used in this investigation are described in Table 1.

**Table 1:** Variables chosen in the study.

Variable	Type	Measuring Unit	Categories	Obtention
*Age*	Independent	Years		Age of the patient recorded on the medical records
	Quantitative			
	Continuous			
*Age group*	Independent	Dimensionless	1 = 18–29 years old	Assignation to a group depending on age
	Qualitative		2 = 30–39 years old	
	Ordinal		3 = 40–49 years old	
			4 = 50< years old	
*Diagnosis*	Independent	Dimensionless	1 = Cervical sprain grade I (ICD-11: NA 23.4)	Diagnosis written in the medical records of the patient correlated with ICD11 [2]
			2 = Cervical sprain grade II (ICD-11: NA 23.4)	
			3 = Cervical sprain grade III (ICD-11: NA 23.4)	
	Qualitative			
	Nominal		4 = Cervicalgia (ICD-11: ME 84.0)	
			5 = Whiplash (ICD-11: NA 23.4)	
			6 = Others (ICD-11: NA 23.4Z)	
*Normalized Flexion-extension ROM*	Independent	Percentage		Normalization of ROM against reference values
	Quantitative			
	Continuous			
*Normalized Lateral Bending ROM*	Independent	Percentage		Normalization of ROM against reference values
	Quantitative			
	Continuous			
*Normalized Rotation ROM*	Independent	Percentage		Normalization of ROM against reference values
	Quantitative			
	Continuous			
*Neck Functional Holistic Analysis Score (NFHAS)*	Dependent	Percentage		NFHAS formula
	Quantitative			
	Continuous			
*NFHAS Type*	Dependent	Dimensionless	1 = Type I, Cluster Centroid = 91.7172	K-means algorithm
			2 = Type II, Cluster Centroid = 72.4324	
	Qualitative		3 = Type III, Cluster Centroid = 56.6505	
	Discrete		4 = Type IV, Cluster Centroid = 38.7712	
			5 = Type V, Cluster Centroid = 17.8583	

Five categories of medical diagnoses were identified in the sample, with an additional “others” category for diagnoses that could not be contained in the previous five. The ICD-11 code is included to link the individual diagnoses with the global reference [2]. Diagnoses where matched to the closest term on ICD-11, the investigators did not reevaluate the diagnose of any patient. All the diagnoses used were the ones given by medical doctors during routine care.

The normalized ROM values for each plane of motion where obtained using the following equations:

$$\begin{array}{l} ROM>ROM_{\mathrm{Normative}}+SD_{\mathrm{Normative}}\to nROM=\frac{ROM}{ROM_{\mathrm{Normative}}+SD_{\mathrm{Normative}}}\times 100\\ ROM<ROM_{\mathrm{Normative}}-SD_{\mathrm{Normative}}\to nROM=\frac{ROM}{ROM_{\mathrm{Normative}}-SD_{\mathrm{Normative}}}\times 100\\ ROM_{\mathrm{Normative}}-SD_{\mathrm{Normative}}\leq ROM\leq ROM_{\mathrm{Normative}}+SD_{\mathrm{Normative}}\to nROM=100\% \end{array}$$

Where ROM represents the full mean range of motion of each plane of motion, SD the standard deviation and nROM the normalized ROM. Normative data was obtained from the work of Swinkels et al. [30].

The values of the nROM (FE, LB, RT) are employed as vertices of a polygon located in a cartesian coordinate system. Since the coordinate system axis are orthogonal to each other, the distance between the vertices can be calculated using the Pythagoras theorem. Each of the ROM values was placed in the coordinate system as: (FE, 0, 0), (0, LB, 0), (0, 0, RT). The equation to calculate the sides of this polygon is:

$$D_{ROM_{a},ROM_{b}}=\sqrt{(x_{ROMa}-x_{ROMb}{)}^{2}+(y_{ROMa}-y_{ROMb}{)}^{2}+(z_{ROMa}-z_{ROMb}{)}^{2}}$$

Where *a* and *b* are any two of the ROM with their *x*, *y* and *z* coordinates. The letter *D* represents the distance between two ROM vertices.

With the three sides obtained, the area of the polygon can be calculated using Heron’s formula:

$$A=\sqrt{s(s-a)(s-b)(s-c)}$$

Where *a*, *b* and *c* are the sides of the polygon, and *s* represents the semiperimeter calculated by:

$$s=\frac{a+b+c}{2}$$

The index created to encompass the ROM of all the three planes (FE, LB, RT) is the Neck Functional Holistic Analysis Score (NFHAS). This index is calculated by dividing the area created with the value of the three ROM (FE, LB, RT) by the area of the regular triangle that would be obtained with a perfect score of 100% in all three ROM.

$$NFHAS=\frac{A}{A_{100\%}}\times 100$$

The clustering of the patients was performed with MATLAB 2018b with the k-means function. This function was used to iteratively obtain 20 clusters. Within cluster variance helped to determine the final optimal number of clusters. Silhouette analysis confirmed the decision of the most appropriate number of clusters.

### Statistical analysis

IBM SPSS, Version 20.0 (IBM Corp. Released 2011. IBM SPSS Statistics for Windows, Version 20.0. Armonk, NY: IBM Corp.) was used for the statistical analysis. Homoscedasticity of the data was checked using the Kolmogorov-Smirnov test. None of the studied variables followed a normal distribution according to this test.

Two different classifications were tested in this investigation to find differences in the impairment of the patients. The first classified patients using classical diagnoses, the second classified the patients according to the NFHAS. The ROM of the created groups was used to assess the differences.

Statistical inference was made using Kruskal-Wallis test. The α value was set at 0.05. In the event that statistically relevant differences were encountered, post-hoc comparisons were made using Mann-Whitney’s U test. Alfa value was adjusted by a Bonferroni correction. This correction is made with the following formula:

$$\alpha_{corrected}=\alpha /m$$

Where the *m* value is the number of tested hypothesis and *α* is the original significance level.

Effect sizes were presented by means of *ηp*^*2*^ for the Kruskal-Wallis H test, and *r* in the case of Mann-Whitney’s U [31–33].

Spearman’s rank-order correlation was used to test the relationship of age with every ROM. It was also used to test the relationship of age and every ROM with the NFHAS.

## Results

### Descriptive statistics

A total of 772 patients were included in this research. In order to have a balanced sampling, researchers requested that this database was a gender-balanced sample [34]. Descriptive statistics of the analyzed variables can be seen in Table 2. Young people suffer more accidents; therefore, the skewness observed in age answers to the natural behavior of the phenomenon. The skewness of the ROM data is conditioned by the natural limit of ROM since few people present values larger than 100% of the normative data interval.

**Table 2:** Descriptive statistics of the normalized ROM values and the age of the patients.

N = 772	Statistic	Value
**Age**	Mean	39.82
	Confidence Interval 95%	38.83–40.82
	Median	38.00
	Standard deviation	14.07
	Minimum	18.00
	Maximum	87.00
	Interquartile range	69.00
	Skewness	0.608
**FE ROM**	Mean	72.35
	Confidence Interval 95%	70.84–73.86
	Median	76.05
	Standard deviation	21.32
	Minimum	6.02
	Maximum	110.45
	Interquartile range	104.43
	Skewness	-0.25
**LB ROM**	Mean	76.02
	Confidence Interval 95%	74,56–77,47
	Median	78.94
	Standard deviation	20.62
	Minimum	10.58
	Maximum	111.10
	Interquartile range	100.52
	Skewness	-0.15
**RT ROM**	Mean	72.57
	Confidence Interval 95%	71,06–74,09
	Median	77.04
	Standard deviation	21.48
	Minimum	0.00
	Maximum	108.03
	Interquartile range	108.03
	Skewness	-0.47

Median age was 38 years old with an inter quartile range (*IQR)* of 20. There are 27.2% of patients between 18–29, 27.2% of patients between 30–39, 21.6% between 40–49% and 24% over 50 years old. The data was positively skewed because of the higher incidence of neck injuries due to a traffic car accident amongst young people [7].

Normalized values for the ROM where obtained with reference values from the matching age interval [30]. Median value for the normalized flexion-extension is 76.05% with an *IQR* of 30.83, median value for the normalized lateral bending is 78.94^o^ and an *IQR* of 29.94; median value for the normalized rotation is 77.04^o^ and an *IQR* of 30.74.

### Analysis of the groups defined by classical diagnosis

According to the ICD-11 five different types of classical diagnoses were identified plus the extra category labeled as others. A 29.6% of patients were diagnosed with cervical sprain (ICD-11:NA23.4), those where further stratified into grades I (26.6%), grade II (1.7%) and grade III (1.3%). A 60.1% of patients were diagnosed with cervicalgia (ICD-11:ME84.0). Only 2.7% of patients were identified with WAD (ICD-11:NA23.4). Finally, 7.6% of the diagnoses had to be grouped into a various category (ICD-11:NA23.4Z) due to the lack of resemblance to any item in the ICD-11.

The Kruskal-Wallis H test was applied to compare the age and ROM from the different groups created by medical diagnosis. This test ranks the variables and compares the mean ranks of these variables between the different groups. The comparison of the ranks made by the test can be seen in Table 3. The distribution of the patients is asymmetric, thus reflecting a possible overdiagnosis of some pathologies and an underdiagnosis of others.

**Table 3:** Mean ranks of the classical diagnosis statistical hypothesis testing.

N = 772	Classical Diagnosis	N	Mean Rank
**Age**	Grade I sprain	205	360,01
	Grade II sprain	13	507,19
	Grade III sprain	10	393,15
	Cervicalgia	464	392,69
	Whiplash	21	423,98
	Others	59	388,76
**FE ROM**	Grade I sprain	205	412,73
	Grade II sprain	13	286,15
	Grade III sprain	10	456,05
	Cervicalgia	464	380,01
	Whiplash	21	312,29
	Others	59	383,13
**LB ROM**	Grade I sprain	205	419,02
	Grade II sprain	13	313,35
	Grade III sprain	10	403,55
	Cervicalgia	464	372,30
	Whiplash	21	364,90
	Others	59	406,08
**RT ROM**	Grade I sprain	205	410,23
	Grade II sprain	13	392,65
	Grade III sprain	10	443,25
	Cervicalgia	464	378,00
	Whiplash	21	260,71
	Others	59	404,73

The Kruskal-Wallis H test shows that there was no statistical difference in the age or ROM values of the patients depending on the medical diagnosis: age X^2^(5) = 7.670, p = 0.175; flexion-extension ROM X^2^(5) = 9.179,p = 0.102; lateral bending ROM X^2^(5) = 8.372, p = 0.137; rotation ROM X^2^(5) = 10.731, p = 0.057. Mean ranks are depicted in Table 1. The effect size (ηp^2^) of the differences between groups regarding ROM was: FE = 0.01, LB = 0.01 and RT = 0.01.

Spearman’s rank-order correlation was run to determine the relationships of every ROM and age. Between age and ROM, only the LB showed a significant correlation with r_s =_ -0.81 and p = 0.024. however, all ROM showed moderate positive correlations between themselves. The FE with the LB resulted in a r_s =_ 0.54 and p = 0.0005 FE with RT resulted in a r_s =_ 0.65 and p = 0.0005. The LB with RT resulted in a r_s_ = 0.56 and p = 0.0005.

### Clustering based on functional results

**Fig 2:**
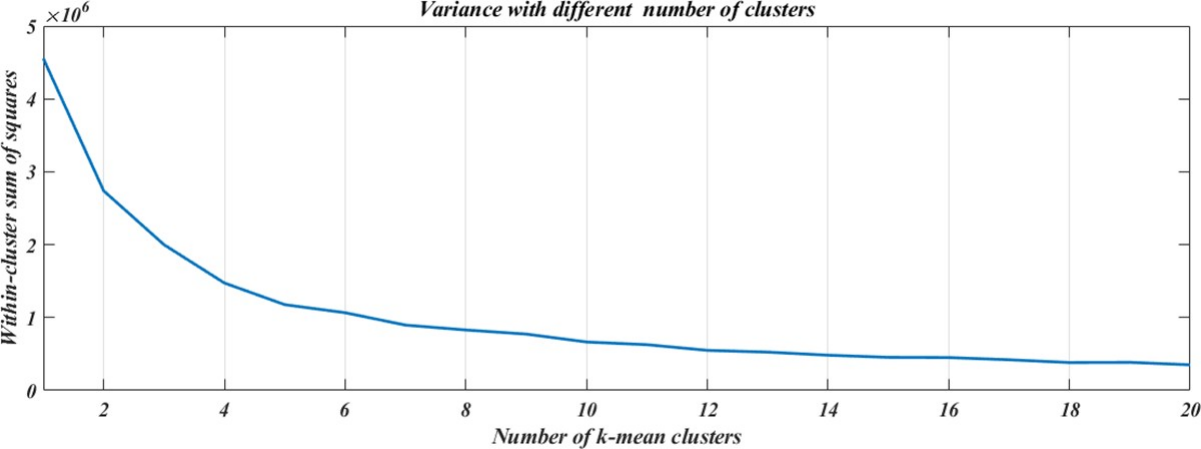
Number of clusters. The diminution of the within cluster variance relents beyond the use of 5 clusters. A more clinically relevant staging would not be obtained by using 6 instead of 5 clusters. Reducing the number of clusters to 4 would result in a grouping of patients with clinically different presentations.

Within cluster variance was calculated from having just 1 cluster, up to 20 (Fig 2). By applying the elbow technique, two optimal options were obtained: 5 or 6 clusters. When using 5 clusters, the sum of squares equaled 1.177x10^6^, while with 6 clusters the sum of squares results in 1.066x10^6^, which results in a difference of just 0.111x10^6^. This difference in variance is smaller than the one previously achieved by increasing the number of clusters. When performing a silhouette analysis, the use of 5 clusters resulted in an average silhouette value of 0.7236, while the average value of 6 clusters was 0.6949. These results suggested choosing 5 groups instead of 6.

With the 5 groups, the different clinical stages of ROM impairment in neck injury are as follow:

- NFHAS type I, mean NFHAS of 91.7% with *SD* 5.89, normalized FE mean value 95.6% with *SD* 5.53, normalized LB mean value of 96.5% and *SD* 6.01, normalized RT mean value of 94.96% and *SD* 6.11. The number of patients included in this cluster is 140.
- NFHAS type II, mean NFHAS of 72.43% with *SD* 4.76, normalized FE mean value 83.46% with *SD* 10.3, normalized LB mean value of 86.79%% and *SD* 11.67, normalized RT mean value of 84.79% and *SD* 9.77. The number of patients included in this cluster is 194.
- NFHAS type III, mean NFHAS of 56.6% with *SD* 4.74, normalized FE mean value 74.28% with *SD* 11.86, normalized LB mean value of 76.9% and *SD* 13.22, normalized RT mean value of 74.21% and *SD* 11.32. The number of patients included in this cluster is 174.
- NFHAS type IV, mean NFHAS of 38.77% with *SD* 5.39, normalized FE mean value 59.75% with *SD* 12.91, normalized LB mean value of 65.84% and *SD* 14.63, normalized RT mean value of 60.32% and *SD* 14.42. The number of patients included in this cluster is 145.
- NFHAS type V, mean NFHAS of 17.85% with *SD* 6.91, normalized FE mean value 39.35% with *SD* 14.26, normalized LB mean value of 45.47% and *SD* 15.38, normalized RT mean value of 38.86% and *SD* 14.07. The number of patients included in this cluster is 118.

**Fig 3:**
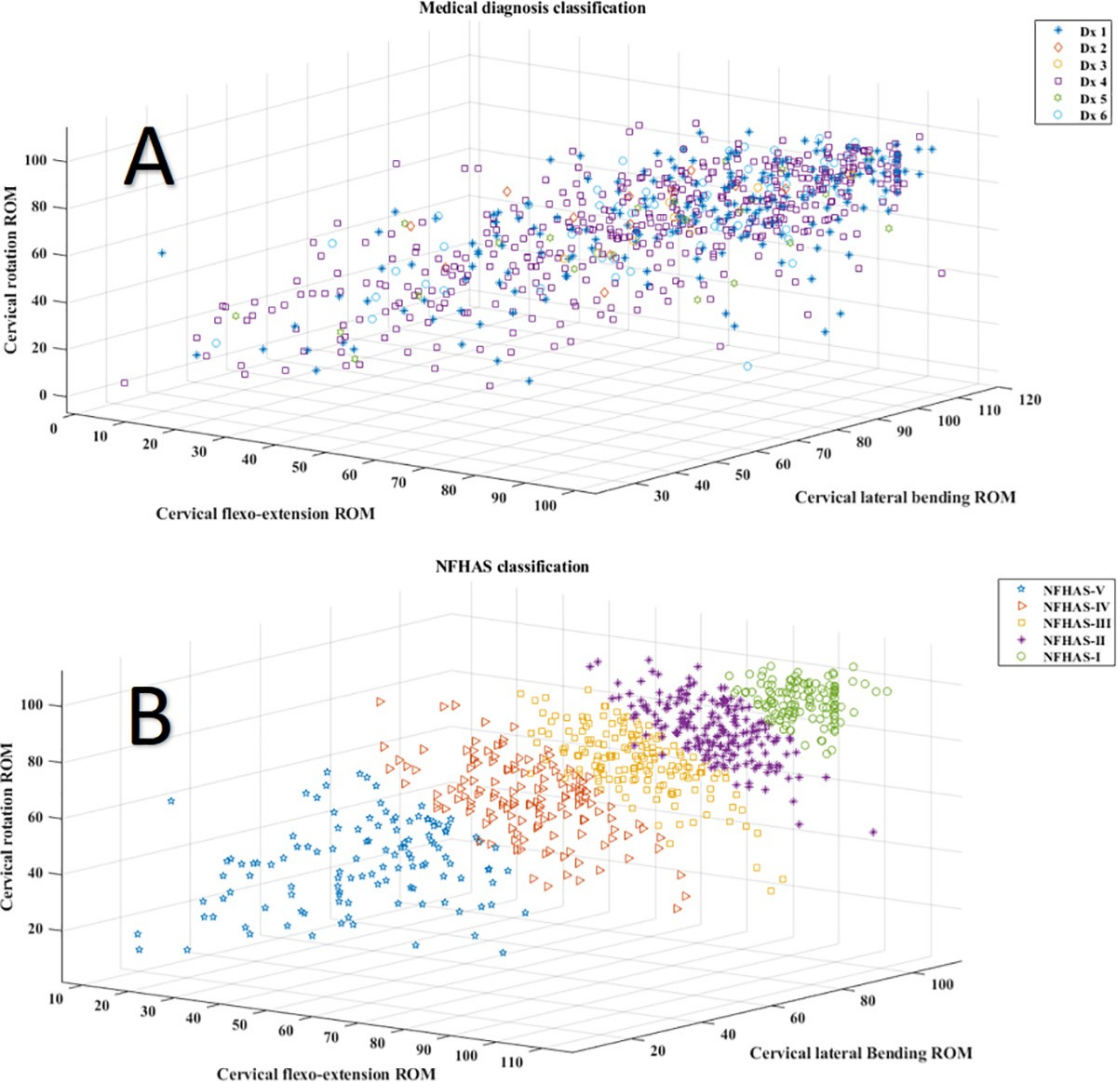
Classification with classical diagnosis vs NFHAS. Classification using the NFHAS provides clearly defined groups that follow a direct distribution according to ROM values. Panel (A) depicts the ROM values of each patient in a 3D space and the color represents the classical diagnosis received. Panel (B) shows the segregation of patients according to their functional ROM values.

The Fig 3 shows and compares the results of classifying the patients both by classical diagnosis and the NFHAS categories.

The Kruskal-Wallis H test shows that there was a statistically relevant difference between the ROM values and NFHAS of the patients depending on the cluster in which they were included: FE X^2^(4) = 551.59, p = 0.0005; LB ROM X^2^(4) = 484.58, p = 0.0005; RT ROM X^2^(4) = 557.14, p = 0.0005; NFHAS X^2^(4) = 737.41, p = 0.0005. Mean ranks are shown in Table 1. The age showed no statistical difference between groups: age X^2^(4) = 3.67, p = 0.452. Effect size (ηp^2)^ of the differences was: FE = 0.76, LB = 0.68, RT = 0.76 and NFHAS = 0.96.

Post-hoc tests with a Bonferroni adjustment of α = 0.05/10 = 0.005 on the ROM and NFHAS using Mann-Whitney’s U test are shown in Table 4.

**Table 4:** Post-hoc analysis of the functional outcomes of the NFHAS groups.

Mann-Whitney’s U test results			
	FE		
	U	p-value	Effect size (r)
**NFHAS type I vs NFHAS type II**	4363	0.0005*	0.58
**NFHAS type I vs NFHAS type III**	1107	0.0005*	0.78
**NFHAS type I vs NFHAS type IV**	53	0.0005*	**0.86**
**NFHAS type I vs NFHAS type V**	1	0.0005*	**0.86**
**NFHAS type II vs NFHAS type III**	9574	0.0005*	0.37
**NFHAS type II vs NFHAS type IV**	2190	0.0005*	0.72
**NFHAS type II vs NFHAS type V**	160	0.0005*	0.79
**NFHAS type III vs NFHAS type IV**	5169	0.0005*	0.5
**NFHAS type III vs NFHAS type V**	664.5	0.0005*	0.79
**NFHAS type IV vs NFHAS type V**	2485.5	0.0005*	0.61
	**LB**		
	**U**	**p-value**	**Effect size (r)**
**NFHAS type I vs NFHAS type II**	6064	0.0005*	0.47
**NFHAS type I vs NFHAS type III**	2083	0.0005*	0.71
**NFHAS type I vs NFHAS type IV**	523.5	0.0005*	**0.82**
**NFHAS type I vs NFHAS type V**	10	0.0005*	**0.86**
**NFHAS type II vs NFHAS type III**	9388.5	0.0005*	0.38
**NFHAS type II vs NFHAS type IV**	3507	0.0005*	0.64
**NFHAS type II vs NFHAS type V**	471.5	0.0005*	0.77
**NFHAS type III vs NFHAS type IV**	7113.5	0.0005*	0.37
**NFHAS type III vs NFHAS type V**	1319.5	0.0005*	0.74
**NFHAS type IV vs NFHAS type V**	2907.5	0.0005*	0.56
	**RT**		
	**U**	**p-value**	**Effect size (r)**
**NFHAS type I vs NFHAS type II**	5042	0.0005*	0.53
**NFHAS type I vs NFHAS type III**	1057	0.0005*	0.78
**NFHAS type I vs NFHAS type IV**	172	0.0005*	**0.85**
**NFHAS type I vs NFHAS type V**	2	0.0005*	**0.86**
**NFHAS type II vs NFHAS type III**	7706	0.0005*	0.46
**NFHAS type II vs NFHAS type IV**	2114.5	0.0005*	0.72
**NFHAS type II vs NFHAS type V**	122	0.0005*	0.79
**NFHAS type III vs NFHAS type IV**	5.383	0.0005*	0.49
**NFHAS type III vs NFHAS type V**	600	0.0005*	0.79
**NFHAS type IV vs NFHAS type V**	2341.5	0.0005*	0.62
	**NFHAS**		
	**U**	**p-value**	**Effect size (r)**
**NFHAS type I vs NFHAS type II**	0.000	0.0005*	**0.85**
**NFHAS type I vs NFHAS type III**	0.000	0.0005*	**0.85**
**NFHAS type I vs NFHAS type IV**	0.000	0.0005*	**0.86**
**NFHAS type I vs NFHAS type V**	0.000	0.0005*	**0.86**
**NFHAS type II vs NFHAS type III**	0.000	0.0005*	**0.86**
**NFHAS type II vs NFHAS type IV**	0.000	0.0005*	**0.85**
**NFHAS type II vs NFHAS type V**	0.000	0.0005*	**0.8**
**NFHAS type III vs NFHAS type IV**	0.000	0.0005*	**0.86**
**NFHAS type III vs NFHAS type V**	0.000	0.0005*	**0.84**
**NFHAS type IV vs NFHAS type V**	0.000	0.00005*	**0.85**

*Significance is set at p<0.005.

Bold effect sizes present a large effect according to Cohen.

All the post-hoc comparisons reflect statistical differences between the groups. Effect sizes are lower between adjacent groups. All the NFHAS values of each group present a large effect size of the difference.

Spearman’s rank-order correlation was run to determine the relationship of the NFHAS with every ROM (FE, LB, RT) and the age of the patients. There was no significative correlation between NFHAS and age r_s_ = -0.37, p = 0.308. On the other hand, strong and significant positive correlations were found for every ROM with the NFHAS. Results for the FE and NFHAS were r_s_ = 0.85, p = 0.0005, for the LB and NFHAS r_s_ = 0.81, p = 0.0005 and for the RT and NFHAS r_s_^=^ 0.86, p = 0.0005.

## Discussion

### The NFHAS relation to the movement

The neck movements of daily living tasks rarely involve just one plane of motion [35] or a single joint. These daily living tasks depend on available range of motion, strength, and appropriate motor control. When the motion of the neck is evaluated, addressing three separate planes of motion greatly increases the difficulty of determining the overall degree of ROM impairment.

The normalized values of the ROM present high correlation coefficients between themselves. Therefore, a combination of them in a single value can facilitate the evaluation of the global range of motion. One option to join all the information into a single value would be to use arithmetical averages; however, these are less robust in the presence of outliers. The implementation of the NFHAS mitigates the effect of outliers on the interpretation of ROM impairment.

Furthermore, Spearman’s rank-order correlation analysis of the NFHAS with the original normalized ROM demonstrates a high correspondence. The NFHAS fluctuates consistently with the variations of ROM in each plane. This relationship shows that the NFHAS provides a full picture of the overall ROM limitation and, therefore, can be used to stage the patient into degrees of ROM impairment.

### The classical diagnoses as stages of movement impairment

Health professionals use classical diagnoses to explain the problems experienced by the victim of a traffic car accident. It was within the scope of this research to check whether the classical diagnoses for the neck injuries are given depending on the severity of signs assessed with the clinical exploration. Inference statistics showed that there was no difference in the ROM impairment between groups with different diagnoses. The scatter plot presented in panel (A) of Fig 2 shows that the multiple diagnoses do not clearly distinguish patients with different impairments of movement. If the classical diagnoses had any relation to the impairment, some clustering should be observed in the data. However, patients with poor results can be seen clustered together with others that have milder ROM loses. The results portraited in Fig 2 panel (A) even show that diagnoses 1, 2 and 3, which are cervical sprains with different categories of severity, do not display statistically relevant differences. There are patients with all levels of impairment inside each cluster. The low effect sizes recovered using the ηp^2^ also suggest that there is no relationship between giving a specific diagnosis and the degree of ROM impairment. Since the ROM integrity will later be used to decide the best treatment and the end of rehabilitation, it would be desirable to have a true reflection of the impairment severity in the diagnosis. Based on these results, classical diagnoses lack information regarding the degree of the patient’s injury. This uncertainty in the assignment of a diagnosis matching the actual capabilities of the patient empowers the “cured by a verdict” myth present in the insurance environment. The way the patients are diagnosed in each country affects the global consideration of the pathology and, therefore, might predispose health professionals to face the patient with mistrust [36]. Guidelines for the assessment of the WAD advise to objectively quantify the signs of the patient with the objective of correctly staging them and proposing the best therapeutic options [37]. It seems, however, that the staging of the patient according to objective signs is not done on a basis in the gathered sample of cases. The degree of impairment should be in combination with the QTF classification to give a more complete overview of the patient’s health. Current guidelines focus mainly on patient reported measures [38] but could benefit from the addition of objective measures in their workflows.

### The NFHAS as clustering tool

Since the classical diagnosis of neck injuries does not have relation to the degree of impairment, the NFHAS was proposed as a quantitative value to perform clustering.

Using the K-means algorithm, 5 main groups were generated. These groups demonstrate statistically relevant differences between them and can allocate different types of impairments. The key differentiating characteristics do not overlap between groups, which indicates a good classification system [39]. Study of the effect sizes reveals that differences between adjacent groups have a low effect, while for groups that are further apart the effect size of the differences becomes moderate to high. It is important to notice that the effect size of the comparison is always high regarding the NFHAS. The associated ROM values vary in a lower proportion between adjacent groups. While the global movement of the patient is highly different between groups, individual ROM values show smaller differences between adjacent groups.

The grading given to the patient is inverse to the NFHAS value. NFHAS type I patients have greater ROM than NFHAS type V patients. This grading of the patients is highly correlated to all the original ROM normalized values and the NFHAS score. The number of stages was decided using the elbow technique, the silhouette analysis, and the clinical meaning of the number of stages. There is not much difference in the within clusters sum of squares between 5 or 6 clusters, nevertheless, there is a clearly lower slope beyond 6 clusters. Silhouette analysis had a greater mean value with 5 clusters than with 6. However, the final decision of choosing 5 clusters answers to the clinical relevance of having an additional cluster. When 6 clusters were made, the division did not give a sufficiently different group regarding impairment. The classification would not benefit from an additional staging of these patients. With 5 groups, patients inside group I are mainly over 90% of the total normalized ROM in every plane, and therefore can be considered as not ROM limited; type II patients have a mild ROM loss; type III a moderate ROM loss; type IV a grave ROM loss and type V a severe loss of ROM. This staging is like the one already used in the decision of the degree of final impairment. The QTF also identifies five categories with increasing severity; however, their grading just considers three categories as uncomplicated damage to soft tissues. This widely used classification only matches the degree of severity of the signs with the staging of the patient on a qualitative basis rather than a quantitative one. This makes possible that a patient with low disability and no neurological signs is classified in the same group as one with high disability and no neurological signs either [9].

A study in patients with low back pain had similar results when staging their patients according to magnetic resonance findings. The study reports a classification where they had one group labeled as ‘no or few findings’, a second group as ‘mild spinal degeneration’, a third group as ‘moderate/severe spinal degeneration’, a fourth group as ‘moderate/severe spinal degeneration and mild sacroiliac joint findings’, and a fifth group as ‘mild spinal degeneration and moderate/severe sacroiliac joint findings’ [40]. It seems appropriate that we have 1 group with no injury or patients considered as not significantly injured, and latter groups with scaling seriousness. The lower bound of the first group lies at 85% of the global functionality, which equates to a mean 90% of the ROM in each plane of motion. A study performed in a WAD population using the NDI identified the 15% of disability as the optimal cutoff for differentiating between real injured and not injured. Although the NDI reflects self-reported disability and not the objective ROM exploration, it is interesting to see that their cutoff matches numerically the one we proposed for the NFHAS type I [41]. Even more, some literature reports that 29 to 38% of the individuals exposed to rear end low energy impacts present symptoms of minimal severity that would disappear within 24 hours of the impact [42]. None of the patients considered in the present study was measured before 48 hours of the impact, since they had to be attended first in the emergency room and later be visited by a physician who prescribed the biomechanical assessment.

Having a more appropriate staging of impairments, like the one proposed, may allow for a proper modelling of the expected outcome of each group depending on the treatments applied. By clustering the neck injured by their ROM impairment, the quality and specificity of the treatments offered to these groups of patients can be improved [39].

### Limitations

It is difficult to gather equal numbers of patients with every diagnosis, so the first limitation is the extreme asymmetry of the groups depending on the clinical diagnosis. This investigation has also highlighted that there is a growing trend to include neck injured patients into the cervicalgia generic diagnosis more than in any other. The diagnosis of the patients was not controlled by the investigation team, and there was no interaction between the investigation team and any of the medical doctors that prescribed those diagnoses, therefore, it only reflects the situation. However, we do not expect that the results would change substantially if we had matching groups for every diagnosis.

Another limitation is that the NFHAS only contains information about the ROM. In prospect investigations, more relevant attributes related to function should be evaluated to be included in the NFHAS and improve the classification. Correlating information of pain level, strength, psychosocial factors, or the function capability of other extremities (such as the upper limb) with the NFHAS could further improve the insight on the WAD pathology.

As it was out of the scope of this paper, no longitudinal assessment of these patients was made. In future studies, patients into each NFHAS group must be examined for their response to rehabilitation. Studying if the groups respond differently to rehabilitation can help in the management of these patients.

Patients are appointed with a medical doctor within ten days of the traffic car accident, however, the information of the exact number of days since the accident was not controlled. This investigation identified degrees of injury that could be present at any stage of the injury, so not having controlled the number of days from the accident should not affect the results substantially. However, in future longitudinal investigations, the information on the time passed since the accident could have influence on the patient’s recovery and should be therefore gathered.

## Conclusions

Classical cervical neck pathology diagnoses are not sensitive enough to correctly stage patients according to their impairment. The NFHAS is directly correlated to the available ROM of the patient. The NFHAS serves as a good tool for the classification of patients into meaningful groups according to movement impairment.
